# Changes in the MicroRNA Profile Observed in the Subcutaneous Adipose Tissue of Obese Patients after Laparoscopic Adjustable Gastric Banding

**DOI:** 10.1155/2017/6754734

**Published:** 2017-03-13

**Authors:** Carmela Nardelli, Laura Iaffaldano, Vincenzo Pilone, Giuseppe Labruna, Maddalena Ferrigno, Nicola Carlomagno, Concetta Anna Dodaro, Pietro Forestieri, Pasqualina Buono, Francesco Salvatore, Lucia Sacchetti

**Affiliations:** ^1^Dipartimento di Medicina Molecolare e Biotecnologie Mediche, Università di Napoli Federico II, Via S. Pansini 5, 80131 Naples, Italy; ^2^CEINGE-Biotecnologie Avanzate Scarl, Via G. Salvatore 486, 80145 Naples, Italy; ^3^Dipartimento di Medicina e Chirurgia, Università di Salerno, Via Giovanni Paolo II 132, Fisciano, 84084 Salerno, Italy; ^4^IRCCS SDN-Istituto di Ricerca Diagnostica e Nucleare, Via Gianturco 113, 80100 Naples, Italy; ^5^Dipartimento di Scienze Biomediche Avanzate, Università di Napoli Federico II, Via S. Pansini 5, 80131 Naples, Italy; ^6^Dipartimento di Medicina Clinica e Chirurgia, Università di Napoli Federico II, Via S. Pansini 5, 80131 Naples, Italy; ^7^Dipartimento Scienze Motorie e del Benessere, Università di Napoli Parthenope, Via Amm. F. Acton 38, 80133 Naples, Italy

## Abstract

*Background*. Laparoscopic adjustable gastric banding (LAGB) results in significant lasting weight loss and improved metabolism in obese patients. To evaluate whether epigenetic factors could concur to these benefits, we investigated the subcutaneous adipose tissue (SAT) microRNA (miRNA) profile before (T0) and three years (T1) after LAGB in three morbidly obese women.* Case Reports*. SAT miRNA profiling, evaluated by TaqMan Array, showed four downexpressed (miR-519d, miR-299-5p, miR-212, and miR-671-3p) and two upexpressed (miR-370 and miR-487a) miRNAs at T1 versus T0. Bioinformatics predicted that these miRNAs regulate genes belonging to pathways associated with the cytoskeleton, inflammation, and metabolism. Western blot analysis showed that PPAR-alpha, which is the target gene of miR-519d, increased after LAGB, thereby suggesting an improvement in SAT lipid metabolism. Accordingly, the number and diameter of adipocytes were significantly higher and lower, respectively, at T1 versus T0. Bioinformatics predicted that the decreased levels of miR-212, miR-299-5p, and miR-671-3p at T1 concur in reducing SAT inflammation.* Conclusion*. We show that the miRNA profile changes after LAGB. This finding, although obtained in only three cases, suggests that this epigenetic mechanism, by regulating the expression of genes involved in inflammation and lipid metabolism, could concur to improve SAT functionality in postoperative obese patients.

## 1. Introduction

Obesity is a major health problem worldwide [1]. Besides lifestyle interventions and pharmacotherapy, significant, lasting weight loss in obese patients can be obtained with laparoscopic adjustable gastric binding (LAGB) surgery [2, 3]. This procedure is usually applied in morbidly obese patients (BMI greater than 40 kg/m^2^) and/or if obesity is associated with comorbidities, such as diabetes or cardiovascular diseases [4]. We previously reported that LAGB also improved metabolism, in terms of inflammation, insulin resistance, and liver steatosis, three years after LAGB [3]. MicroRNAs (miRNAs) are short (19–22 bp) noncoding RNAs that regulate the expression of mRNA targets mainly by binding their complementary sequences at the 3′ untranslated region (3′UTR) and inhibiting their translation [5]. The miRNA profile in subcutaneous (SAT) and in visceral (VAT) adipose tissues differs between obese and lean subjects [6, 7]. The aim of the present study was to investigate the SAT miRNA profile in three morbidly obese women before (T0) and three years (T1) after LAGB and to evaluate whether miRNAs are involved in post-LAGB metabolic improvement.

## 2. Methods

### 2.1. Patients

Three obese women (mean body mass index [BMI] 42.9 kg/m^2^; mean age 48 years) undergoing LAGB (at T0 and T1) and two lean controls (mean BMI 21.5 kg/m^2^; mean age 37 years) undergoing laparoscopic cholecystectomy entered the study. All subjects gave informed consent to the study, which was performed according to the Helsinki II Declaration and was approved by the Ethics Committee of our School of Medicine.

### 2.2. Serum Determinations

A fasted serum sample was also collected from enrolled subjects, the main biochemical parameters were measured by routine methods; leptin (L) and adiponectin (A) adipokines were measured by ELISA methods and the L/A ratio was calculated.

### 2.3. Adipocytes Size and Number Quantification

Periumbilical bioptic SAT samples were obtained from all subjects. All biopsies were snap-frozen and stored in liquid nitrogen until RNA isolation. The number and diameter of adipocytes (at T0 and T1) were measured as previously reported [3]. Briefly, hematoxylin and eosin stains were used to identify the cellular components and their morphological changes. For each sample, adipocytes were counted in three fields (100 *μ*m each) and their size was measured by two operators; the average of each parameter was then calculated.

### 2.4. RNA Isolation

Total RNA (including miRNAs) was purified from SAT using the mirVana™ miRNA isolation kit (Ambion, Austin, TX, USA), and its concentration was evaluated by NanoDrop® ND-1000 UV-Vis spectrophotometer (NanoDrop Technologies, Wilmington, DE, USA).

### 2.5. miRNAs Expression Profile

TaqMan low density arrays Human MicroRNA Panel v1.0 (Applied Biosystems Inc., Foster City, CA, USA), containing 377 preloaded human miRNA targets and the endogenous control RNU48, were used according to the manufacturer's instructions. RT-PCR was performed with 800 ng of cDNA and the 7900 HT real-time PCR system (Applied Biosystems) as reported elsewhere [7]. miRNA expression was normalized to RNU48 and quantified using the RQ Manager 1.2 software (Applied Biosystems) with the following formula: RQ = 2^−ΔΔCt^, where ΔΔCt = (Ct_obese_[miRNA] − Ct_obese_[RNU48]) − (Ct_calibrator_[miRNA] − Ct_calibrator_[RNU48]). miRNAs whose mean baseline RQ levels were <0.5 (downexpressed) or >2.0 (upexpressed) in all obese patients versus controls were considered differently expressed. We compared the expression of these differently expressed miRNAs with those previously reported in the SAT of other obese cohorts [6, 8–14]. Only miRNAs whose expression trend was confirmed were selected for evaluation after LAGB.

The levels of miR-370 and miR-487a (upexpressed) and miR-519d (downexpressed) were validated by TaqMan miRNA assays (Applied Biosystems) in accordance with the manufacturer's instructions on the 7900 HT real-time PCR system (Applied Biosystems).

### 2.6. PPAR-Alpha Western Blot

Total proteins were extracted, quantified, and separated by 15% sodium dodecyl sulfate-polyacrylamide gel electrophoresis. Western blot analyses of PPAR-*α* (dilution 1 : 400) and actin (dilution 1 : 1000) proteins were performed with antibodies from Santa Cruz Biotechnology (Santa Cruz, CA, USA) [6].

### 2.7. Bioinformatics

The MiRTarBase, STRING, and KEGG databases were used to select the experimentally validated miRNA/target gene interaction, to explore protein-protein interactions and to identify the pathways significantly (*p* < 0.001) deregulated, respectively.

### 2.8. Statistics

Biochemical and clinical data are expressed as mean and standard error of the mean (SEM), whereas miRNA expression data are reported as log_10_ mean RQ and SEM. The Wilcoxon test was used to compare data obtained at T0 and T1. Differences were considered statistically significant at *p* < 0.05. Statistical analyses were carried out with the PASW Statistics (Ver.18; SPSS Inc. Headquarters, Chicago, Ill. USA).

## 3. Results

Leptin levels and the L/A ratio were lower, whereas adiponectin levels were higher, although not significantly, at T1 versus T0 (Table 1). Furthermore, the number of SAT adipocytes was higher and their diameter lower (*p* < 0.05) at T1 versus T0 (Table 1) in all three obese patients. Overall, 68% (257/377) of miRNAs were expressed at T0 in SAT from obese and control subjects. Most of these miRNAs (199/257) were not differentially expressed between obese and lean subjects and were not further investigated, whereas 58 miRNAs were upexpressed (74%, 43/58) or downexpressed (26%, 15/58) in obese versus controls (Table 2).

**Figure 1:**
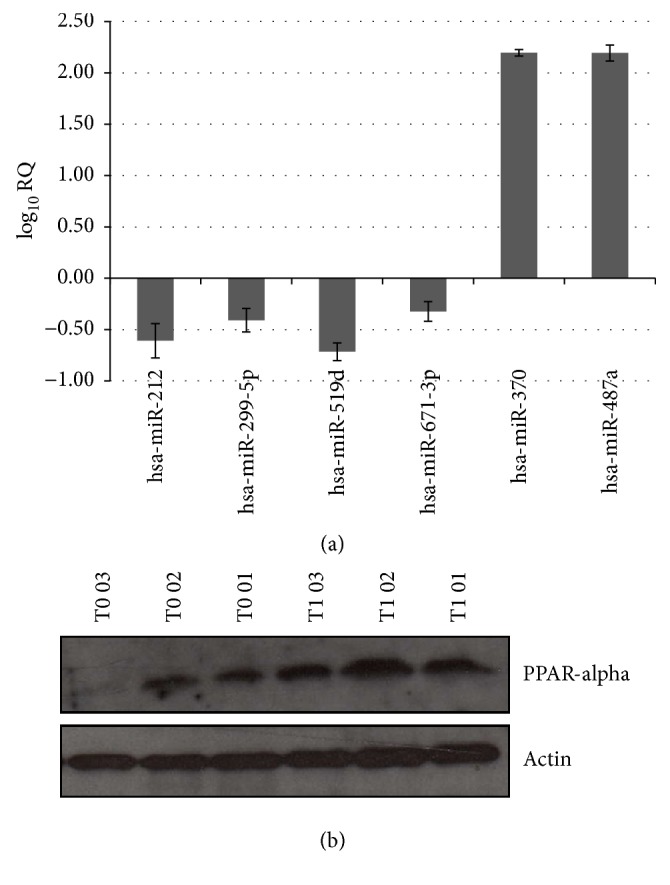
Six miRNAs whose expression changed in subcutaneous adipose tissue (SAT) three years after LAGB (T1) and Western blot evaluation of protein PPAR*α*, which is targeted by miR-519d. (a) Expression levels of 4 downexpressed (miR-212, miR-299-5p, miR-519d, and miR-671-3p) and 2 upexpressed miRNAs (miR-370 and miR-487a) after LAGB (T1 versus T0). MiRNA expression levels are shown as mean log_10_⁡RQ (the miRNA expression values were first normalized to RNU48, after which the relative quantification was calculated as RQ = 2^−ΔΔCt^, where ΔΔCt = [Ct  obese(miRNA) − Ct  obese(RNU48)] – [Ct  calibrator(miRNA) – Ct  calibrator(RNU48)]. (b) Western blot evaluation of protein PPAR-*α* from SAT of 3 obese patients before (T0) and three years (T1) after LAGB.

Table 2 also shows the 31/58 miRNAs (25 upexpressed and 6 downexpressed) previously reported to have a similar obese-associated trend in other cohorts [6, 8–14]. Six of these 31 miRNAs showed post-LAGB changes: miR-519d, miR-299-5p, miR-212, and miR-671-3p were downexpressed at T1 versus T0, and miR-370 and miR-487a were upexpressed at T1 versus T0 (Figure 1(a)). Single RT-PCR assay confirmed miR-370, miR-487a, and miR-519d post-LAGB expression (mean RQ values: 3.54, 3.79, and 0.65, resp., at T1 versus T0). Notably, at Western blot analysis, the expression of protein PPAR-*α*, whose mRNA is targeted by miR-519d [6], was significantly higher (*p* < 0.05) at T1 versus T0 (mean OD/SD: 1.84/0.3 versus 0.72/0.4, resp.) (Figure 1(b)).

**Figure 2:**
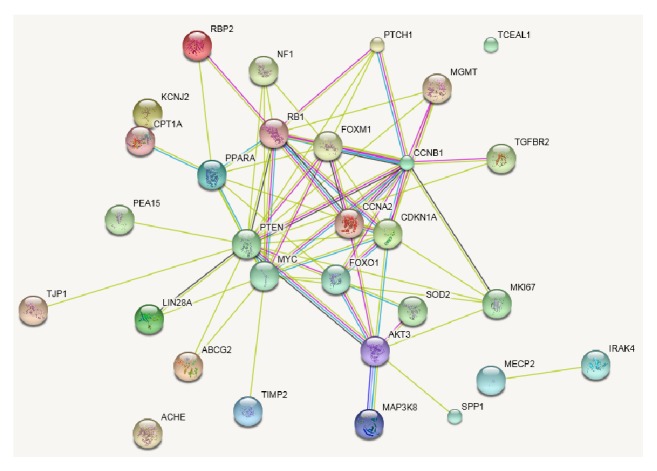
Metabolic pathways predicted to change by our miRNAs after LAGB. Network identified by STRING showing a close interaction among the proteins regulated by the subset of 6 miRNAs. The above 6 miRNAs also targeted the pathways of several proteins in cancer and infection diseases (data not reported).

Bioinformatics predicted that, by targeting several genes, these six miRNAs significantly (*p* < 0.001) regulated interrelated pathways associated with the cytoskeleton, inflammation, and metabolism, that is, FoxO, cell cycle, toll-like receptor, MAPK, AMPK, p53, PI3K-Akt, adipocytokine, tight and adherens junctions, focal adhesion, mTOR, PPAR, TGF-beta, and apoptosis (Table 3 and Figure 2).

## 4. Discussion

In three obese patients, we identified 6 miRNAs whose expression levels were either lower (miR-519d, miR-212, miR-299-5p, and -miR-671-3p) or higher (miR-370 and miRNA-487a) after LAGB. These miRNAs were predicted to regulate pathways involved in obesity or in obesity-related cellular dysfunctions, the most significant being adipocytokine and PPAR signaling, some interrelated metabolic pathways (FoxO, cell cycle, MAPK, AMPK, p53, PI3K-Akt, TGF-beta signaling, and apoptosis), the cytoskeleton, and cell-cell adhesion (focal adhesion and tight and adherens junctions).

We previously found higher miR-519d expression in SAT from another group of obese patients versus lean subjects and demonstrated that it targeted PPAR-*α* mRNA, thereby reducing the level of this protein in SAT and causing lipid accumulation during adipocyte differentiation [6]. Data obtained in PPAR-*α*-null mice showed that this protein is involved in lipid homeostasis [15]. Thus, our finding of lower miR-519d levels and higher PPAR-*α* protein expression in SAT after LAGB, together with the significantly (*p* < 0.05) smaller diameter and higher number of adipocytes at T1 versus T0, suggests that surgery might improve lipid metabolism and adipose tissue functionality also via a miR-519d-mediated mechanism. Accordingly, PPAR-*α* activation was reported to induce removal of intracellular lipids through fatty acid oxidation [15] and to be a part of a regulatory loop that controls the metabolic response of adipose tissue to nutrients and other signals [15]. Furthermore, PPAR-*α* is also a nutritional sensor adapting metabolic homeostasis to energy deprivation; it exerts an anti-inflammatory function and controls also some aspects of the glucose and lipid metabolism in several tissues included the adipose one [16].

The decrease of miR-212, miR-299-5p, and miR-671-3p levels that we found at T1 versus T0 was predicted to be associated with reduced SAT inflammation. This is in line with the findings that (i) miR-212 is directly involved in inflammation and immune processes [17] and (ii) miR-299-5p, by upregulating its target gene SPP1, could promote endocytosis of cell debris and indirectly reduce inflammation [18]. Lastly, an earlier study showed that miR-671-3p expression was lower in visceral fat from obese patients with nonalcoholic steatohepatitis versus obese patients with nonalcoholic fatty liver disease [19] and was related to decreased liver inflammation.

Concerning miR-212, it was demonstrated to be upexpressed in high fat fed mice and its levels measured in liver were downregulated by physical activity [20]. miR-212 positively regulated fatty acids biosynthesis and hepatic storage by acting on fibroblast growth factor 21 (FGF-21) [20]. Interestingly, FGF-21 is a target gene of PPAR-*α* and is involved in cellular response to oxidative stress [21].

Consequently, the reduced SAT expression of both miR-212 and -519d, that we observed in our obese patients after LAGB, could exert a role in reducing oxidative stress and inflammation by acting on the PPAR-*α*-FGF-21 axis. In agreement with this hypothesis, we previously reported a reduced SAT inflammation after LAGB in a larger cohort compared to the present [3] and here we suggest that this effect could be, in part, caused by a miRNA-mediated mechanism.

Finally, although miR-370 and 487a, which were upexpressed after LAGB, were predicted to regulate the expression of genes belonging to several metabolic pathways, their contribution to surgery metabolic improvement, if any, remains to be established.

## 5. Conclusions

In conclusion, although our data were obtained in only three patients (mainly because patients were unwilling to undergo a post-LAGB biopsy), we show that the miRNA profile changes after LAGB. This finding suggests that this epigenetic mechanism, by regulating the expression of genes involved in inflammation and lipid metabolism, could concur to improve SAT functionality in postoperative obese patients.

## Figures and Tables

**Table 1:** Clinical and biochemical parameters measured in three obese females (OB) before (T0) and three years after LAGB (T1).^*∗*^

	OB1		OB2		OB3	
	T0	T1	T0	T1	T0	T1
BMI (kg/m^2^)^*∗∗*^	41.5	28.4	37.2	28.1	50.0	39.5
EWL (%)		71.4		66.7		36.2
Na^+^ (mmol/L)	138.0	143.0	142.0	139.0	143.0	142.0
K^+^ (mmol/L)	4.0	4.3	4.3	4.8	4.7	4.7
Ca^++^ (mg/dL)	10.1	9.3	9.5	10.8	9.2	9.9
Phosphorus (mg/dL)	3.4	3.1	3.7	3.4	2.9	3.6
Fe (*µ*g/dL)	90.0	49.0	145.0	72.0	47.0	85.0
Glucose (mmol/L)	5.7	5.8	4.6	4.8	5.0	4.9
Insulin (mU/L)	6.1	11.3	12.9	3.5	51.9	11.2
HOMA	2.9	1.6	2.6	0.7	11.5	2.4
Cholesterol (mmol/L)	5.0	4.9	5.0	4.8	4.1	4.8
Triglycerides (mmol/L)	0.9	0.8	1.0	0.5	0.8	1.0
AST (U/L)	17.0	15.0	24.0	13.0	24.0	26.0
ALT (U/L)	20.3	11.0	24.0	26.0	23.0	26.0
ALP (U/L)	90.0	74.0	64.0	58.0	112.0	110.0
GGT (U/L)	16.7	11.0	20.0	12.0	15.0	12.0
LDH (U/L)	307.0	375.0	374.0	102.0	335.0	336.0
CHE (U/L)	10037.3	6497.0	11312.0	8383.7	8721.0	8855.0
CK (U/L)	100.0	51.0	128.0	97.7	61.0	54.0
AMS (U/L)	53.0	53.0	39.0	45.0	56.0	78.0
Urea (mmol/L)	0.4	0.5	0.5	0.5	0.7	0.5
Creatinine (*μ*mol/L)	53.0	53.0	61.9	53.0	61.9	70.7
UA (mmol/L)	0.3	0.2	0.3	0.2	0.3	0.3
T-bil (*μ*mol/L)	9.7	7.7	18.1	6.8	6.8	12.1
TP (g/dL)	7.8	7.5	7.7	6.4	8.0	8.3
Albumin (g/dL)	4.2	4.4	4.8	4.5	4.0	4.6
Adiponectin (*µ*g/mL)	9.4	19.5	7.7	15.7	9.9	17.1
Leptin (ng/mL)	43.1	8.5	22.4	12.4	32.2	16.6
Leptin/adiponectin ratio	4.6	0.4	2.9	0.8	3.2	1.0
Adipocyte number^*∗∗*^	31	55	35	48	31	46
Adipocyte diameter (*µ*m)^*∗∗*^	14	8	13	9	14	10

^*∗*^Data from [3]. ^*∗∗*^Statistically significant differences at T0 versus T1 at Wilcoxon's test; *p* < 0.01.

ALP: alkaline phosphatase; ALT: alanine aminotransferase; AMS: amylase; AST: aspartate aminotransferase; BMI: body mass index; CHE: cholinesterase; CK: creatine kinase; EWL: excess weight loss; GGT: *γ*-glutamyl transferase; HOMA: homeostasis model assessment; LDH: lactate dehydrogenase; T-bil: total bilirubin; TP: total proteins; UA: uric acid.

**Table 2:** Differently expressed miRNAs at T0 in obese patients versus normal-weight subjects.

Upexpressed miRNAs	Downexpressed miRNAs
*hsa-let-7a*	hsa-miR-18b
*hsa-let-7d*	hsa-miR-107
hsa-let-7e	*hsa-miR-200a*
*hsa-let-7f*	*hsa-miR-200c*
*hsa-miR-10a*	*hsa-miR-205*
*hsa-miR-21*	hsa-miR-331-5p
*hsa-miR-27b*	hsa-miR-342-5p
*hsa-miR-29c*	*hsa-miR-370*
*hsa-miR-34a*	*hsa-miR-382*
*hsa-miR-92a*	hsa-miR-429
hsa-miR-95	*hsa-miR-487a*
*hsa-miR-99b*	hsa-miR-496
*hsa-miR-100*	hsa-miR-520a-3p
*hsa-miR-125b*	hsa-miR-548c-5p
hsa-miR-128	hsa-miR-615-5p
*hsa-miR-130a*	
*hsa-miR-130b*	
*hsa-miR-145*	
*hsa-miR-146b-5p*	
hsa-miR-190	
*hsa-miR-195*	
hsa-miR-199b-5p	
hsa-miR-204	
*hsa-miR-212*	
*hsa-miR-221*	
*hsa-miR-224*	
hsa-miR-296-5p	
*hsa-miR-299-5p*	
hsa-miR-323-3p	
hsa-miR-328	
hsa-miR-339-5p	
hsa-miR-362-3p	
*hsa-miR-365*	
hsa-miR-494	
*hsa-miR-519d*	
hsa-miR-520g	
hsa-miR-532-3p	
*hsa-miR-652*	
hsa-miR-655	
*hsa-miR-671-3p*	
hsa-miR-708	
hsa-miR-744	
hsa-miR-758	

Italic type indicates miRNAs whose expression trend was similar to those of previous studies [6, 8–14]. These miRNAs were investigated after LAGB.

**Table 3:** Metabolic pathways predicted to change by our miRNAs after LAGB. The most significant pathways (*p* < 0.005) predicted by KEGG to contain genes targeted by the 6 miRNAs whose expression changed three years after LAGB.

KEGG pathways		
Pathway description (ID)	FDR	miRNAs
*FoxO signaling pathway (4068)* AKT3, CCNB1, CDKN1A, FOXO1, PTEN, SOD2, TGFBR2	1.05*e* − 07	miR-519d, miR-212, miR-299-5p, miR-370
*Cell cycle (4110)* CCNA2, CCNB1, CDKN1A, MYC, RB1	4.03*e* − 05	miR-519d, miR-299-5p, miR-212
*Toll-like receptor signaling pathway (4620)* AKT3, IRAK4, MAP3K8, SPP1	0.000363	miR-519d, miR-299-5p, miR-212, miR-370
*MAPK signaling pathway (4010)* AKT3, MAP3K8, MYC, NF1, TGFBR2	0.000567	miR-519d, miR-212, miR-370
*AMPK signaling pathway (4152)* AKT3, CCNA2, CPT1A, FOXO1	0.000567	miR-519d, miR-212, miR-370
*p53 signaling pathway (4115)* CCNB1, CDKN1A, PTEN	0.00167	miR-519d, miR-299-5p, miR-212
*PI3K-Akt signaling pathway (4151)* AKT3, CDKN1A, MYC, PTEN, SPP1	0.00167	miR-519d, miR-299-5p, miR-212
*Adipocytokine signaling pathway (4920)* AKT3, CPT1A, PPARA	0.00167	miR-519d, miR-370
*Tight junction (4530)* AKT3, PTEN, TJP1	0.00821	miR-519d, miR-212
*Focal adhesion (4510)* AKT3, PTEN, SPP1	0.0259	miR-519d, miR-299-5p
*mTOR signaling pathway (4150)* AKT3, PTEN	0.0269	miR-519d
*PPAR signaling pathway (3320)* CPT1A, PPARA	0.0346	miR-370, miR-519d
*Adherens junction (4520)* TGFBR2, TJP1	0.0356	miR-370, miR-212
*TGF-beta signaling pathway (4350)* MYC, TGFBR2	0.0418	miR-212, miR-370
*Apoptosis (4210)* AKT3, IRAK4	0.0483	miR-519d, miR-212
